# Flourishing as a guide to intervention: a national multicenter study of general surgery residents

**DOI:** 10.1007/s44186-022-00014-3

**Published:** 2022-03-31

**Authors:** Anya L. Greenberg, Christy Boscardin, Carter C. Lebares, The General Surgery Research Collaborative on Resident Wellbeing, Celia M. Divino, Jennifer N. Choi, Jennifer E. Hrabe, Julia S. Shelton, Christopher M. Foglia, Varuna M. Sundaram, Brooke Gurland, David A. Spain, Matthew J. Hanlon, Andreas H. Meier, Kelly R. Haisley, Alan E. Harzman, Emily Huang, Jennifer F. Preston, Valentine N. Nfonsam, Taylor S. Riall, Barnard J. A. Palmer, Gregory P. Victorino, Tim R. Donahue, Veronica F. Sullins, Anya L. Greenberg, Kenzo Hirose, Carter C. Lebares, Linda M. Reilly, Kshama R. Jaiswal, Mark R. Nehler, Melissa E. Brunsvold, Daniel E. Kendrick, M. Timothy Nelson, Robert B. Lim, Karen D. Horvath, Lorrie A. Langdale, Rebecca Maine

**Affiliations:** 1 Department of Surgery, University of California San Francisco, San Francisco, CA USA; 2 Department of Medicine, University of California San Francisco, San Francisco, CA USA

**Keywords:** Surgical resident wellbeing, Mindfulness for surgeons, Flourishing, Job strain, Distress, Surgical education

## Abstract

**Purpose:**

Physician wellbeing is critical to maximize patient experience, quality of care, and healthcare value. Objective measures to guide and assess efficacy of interventions in terms of enhanced thriving (as opposed to just decreased pathology) have been limited. Here we provide early data on modifiable targets, potential interventions, and comparative impact.

**Methods:**

In this cross-sectional survey-based study of mixed-level residents at 16 academic General Surgery training programs, gender-identity, race, post-graduate year, and gap years were self-reported. Correlation between our primary outcome variable, flourishing, and measures of resilience (mindfulness, personal accomplishment [PA], workplace support, workplace control) and risk (depression, emotional exhaustion, depersonalization, perceived stress, anxiety, workplace demand) were assessed.

**Results:**

Of 891 recipients, 300 responded (60% non-male, 41% non-white). Flourishing was significantly positively correlated with all measured resilience factors and negatively correlated with all measured risk factors. In multivariable modelling, mindfulness, PA, and workplace support were positively and significantly associated with flourishing, with PA having the strongest resilience effect. Depression and anxiety were negatively and significantly associated with flourishing, with depression having the strongest risk effect.

**Conclusions:**

Our results suggest that interventions that increase mindfulness, workplace support, and PA, as well as those that decrease depression and anxiety may particularly impact flourishing (i.e., global wellbeing) in surgical trainees. These findings provide preliminary guidance on allocation of resources toward wellbeing interventions. In particular, cognitive (i.e., mindfulness) training is a feasible intervention with modest but significant association with flourishing, and potential indirect effects through influence on PA, anxiety and depression.

**Supplementary Information:**

The online version contains supplementary material available at 10.1007/s44186-022-00014-3.

## Background

Physician wellbeing is critical to maximize patient experience, quality of care, and healthcare value [9, 77]. Its absence impacts the private and professional lives [76] of surgeons and trainees and is at the root of the Accreditation Council on Graduate Medical Education (ACGME) mandate for formal wellbeing programs [3]. While addressing pathologies, such as burnout [38], anxiety, depression [48], and suicidality, has been the focus for several decades, only recently have we reframed our goal as optimizing surgeon wellbeing or thriving [23]. A key challenge has been the absence of a measure of global wellbeing with validity evidence in physicians and surgeons. Moreover, despite numerous proposed frameworks and interventions to promote wellbeing [5, 7, 67], objective measures to assess efficacy in terms of enhanced thriving (as opposed to just decreased pathology) have been limited [32, 38, 48]. This has prevented training programs from objectively evaluating and comparing interventions, and hindered evidence-based guidance on how to invest precious resources to truly optimize surgeon wellbeing.

Validity evidence has been demonstrated for flourishing, an established construct of social, emotional, and psychological thriving in non-physicians measured by the Mental Health Continuum-Short Form (MHC-SF; for simplicity referred to herein as MHC) [41], as a global measure of global wellbeing in surgical trainees, regardless of race or gender [53]. This was evidenced by positive correlation between flourishing and known resilience factors (such as mindfulness, personal accomplishment, and perceived workplace support and control) and negative correlation between flourishing and known risk factors (such as depression, burnout, stress, anxiety, and perceived workplace demand). This suggests that interventions targeting these factors alone or in combination may enhance flourishing (i.e., thriving or global wellbeing) in surgical trainees. Given residency programs’ limited financial and human capital resources, it is essential to understand which factors impact flourishing, which of these factors are modifiable, and the relative impact of these factors on flourishing to guide effective implementation decisions.

We explored these questions by surveying mixed-level General Surgery residents nationally using published measures of resilience and risk factors. Our goal was to provide early data on modifiable targets, potential interventions, and magnitude of association, to guide the design, evaluation, and prioritization of future wellbeing initiatives whose efficacy can be objectively measured through their effect on flourishing. Understanding the nuances of these differences will help surgical training programs to identify the highest-impact, most feasible wellbeing interventions.

## Methods

### Study design

An online survey instrument was distributed in January 2021 to all preliminary and categorical General Surgery residents (both clinically active and in research) at 16 ACGME-accredited academic training programs representing Western, Mountain, Central and Eastern regions of the US, ranging in size from 21 to 108 residents.. Participating programs comprise the General Surgery Research Collaborative on Resident Wellbeing, which evolved during the first surge of Coronavirus Disease 2019 due to outreach from the UCSF Center for Mindfulness in Surgery.

Champions at participating programs disseminated the survey to their respective resident bodies, indicating that the survey is anonymous, all questions are optional, and results will only be viewed in aggregate. 891 residents across the 16 programs received the survey, which remained open for six weeks. Survey responses were aggregated and analyzed. Aggregated program-specific data were shared only with programs with response rate of at least 30% to ensure respondent anonymity. Participants who completed the survey optionally submitted a separate survey (to maintain anonymity) with their name and any email address to claim a $5 coffee card for their participation. There was no linking information between the two surveys. The study was approved by UCSF’s institutional review board and informed consent was obtained for all participants.

### Survey instrument

The anonymous survey collected basic demographic information and measured the presence of resilience, which is characterized by high positive emotions, nonreactivity to stressors, and connectedness (as defined by seminal works in the field of resilience science [25, 30, 59, 60, 72, 79] and used in prior studies as proxy measures of wellbeing [12, 48, 51, 52, 64]). The survey further measured distress, characterized by high burnout, stress, anxiety or depressive symptoms (as defined by multiple works exploring distress in surgery and perceived in the literature to be discordant with wellbeing [38, 48]). These Likert scale-based measures, found reliable in our prior work with surgical trainees, were scored according to published methods described hereinafter. Including demographic information, the survey consisted of 77 questions (Appendix 1) and was estimated to take 10 min to complete.

Our primary outcome variable, flourishing, was assessed through the *Mental Health Continuum-Short Form (MHC-SF)*, a 14-item measure of psychosocial wellbeing with a three-factor model reflecting social, emotional, and psychological mental health domains (Appendix 2), with high internal consistency (> 0.80) [42] and supporting literature base of clinical relevance [32]. Similar to standard diagnostic criteria for depression, the MHC-SF items are scored according to the frequency with which respondents experience each symptom of positive mental health. Per convention, categorical designation using this measure is not limited to a specific numeric cut-off. Rather, flourishing represents experiencing high positive functioning and high positive emotions ‘every day’ or ‘almost every day.’ Scores can also be treated continuously [47]. In our work, we use the continuous (i.e., ‘MHC score’) form of this measure.

To assess individual-level resilience and risk factors, we used several published and widely accepted measures used in our prior work. The *Cognitive Affective Mindfulness Scale-Revised (CAMS-R)* is a 10-item measure of both dispositional and trained mindfulness in the form of attention, present-focus, awareness, and acceptance, with internal consistency (0.7–0.74) [26] and a calculated global score, shown to increase with mindfulness training [32]. Higher CAMS-R scores are associated with lower odds of distress in surgical trainees [26, 48]. The *abbreviated Maslach Burnout Inventory (MBI)* is a 9-item validated screen [58] for high emotional exhaustion (EE), depersonalization (DP), and personal accomplishment (PA) (use and scoring described by McManus et al. [63]), each associated with multiple negative sequelae in surgical trainees [24, 45, 48, 51]. *Cohen’s Perceived Stress Scale (PSS)* is a 10-item widely-used measure of stress, with high internal consistency (> 0.80) [17, 65] with normative data for men and women aged 18–34. High PSS scores correlate with cognitive impairment, missed work and disability [66]. *Spielberger’s State Trait Anxiety Index (STAI)* is a 6-item measure of subjective feelings (e.g., apprehension, tension) and autonomic arousal [4, 35, 46, 80, 82] correlated with state anxiety. A cutoff of ≥ 40 used in other studies to denote high anxiety [4, 46]. In surgical trainees, during real-life and simulated trauma scenarios, the STAI has high internal consistency (0.92) [80]. The *Patient Health Questionnaire-8 (PHQ-8)* is an 8-item rigorously evaluated and validated depression screening tool [21] with high internal consistency (0.88) [78]. A total score of > / = 10 correlated is with increased use of clinical resources [74].

Finally, we explored the influence of risk and resilience factors within the workplace through the* Swedish Demand-Control-Support Questionnaire (DCSQ),* which is a 16-item measure of job strain with good internal consistency (0.7–0.85) [71] rooted in Job Demand-Resource theory. Subdomains exist for Demand, Control and Support. High workplace demand and low control are known risks for job strain, while high workplace control and social support are known to decrease risk and mitigate the effects of demand [19, 71]. High subdomain designations are defined as scores within the upper third of the total possible score [70].

### Data analysis

Descriptive statistics were derived to characterize participant characteristics. Counts and percentages were reported for nominal data. Unadjusted correlation between total MHC score and each resilience and risk factor was assessed using linear regression, and then a multivariable model including both resilience and risk factors was performed. Multicollinearity issues were addressed if any variables had a correlation coefficient ≥ 0.7 or a variance inflation factor (VIF) of ≥ 4. In these cases, variables were selected for inclusion based on theoretical and clinical evidence. Partial omega-square standardized effect sizes (i.e., Std. *β*) were estimated to assess the relative impact of each measure in the model. Complete case analysis was used for the multivariable model. The model subsample was compared to the subsample excluded due to missing data. Participant characteristics, including gender, race/ethnicity, and training level were compared between the two groups using Fisher’s exact tests. Hypothesis tests were two-sided, and the significance threshold was set to 0.05. Statistical analyses were performed using SAS version 9.4.

## Results

### Respondents

Three hundred residents (60% non-male, 41% non-white, 57% junior residents) responded to the survey, representing a 34% response rate (Table 1). This is slightly skewed toward non-male and non-white residents as compared to the demographics of the entire body of US General Surgery residents [24].

**Table 1:** Participant characteristics of 300 survey respondents

Characteristic	No. (%)
*Self-reported gender identity*	
Non-male	179 (59.6)
Female	178 (59.3)
Genderqueer/gender nonconforming	1 (0.3)
Transgender man	0 (0.0)
Transgender woman	0 (0.0)
Male	119 (39.7)
Decline to state	2 (0.7)
*Self-reported race/ethnicity*	
White	171 (57.0)
Non-White	123 (41.0)
Asian (includes Asian + White)	70 (23.3)
Latinx (includes Latinx + White)	28 (9.3)
Black/African American (includes Black/African American + White)	11 (3.7)
Other	11 (3.7)
American Indian/Alaska Native (includes American Indian/Alaska Native + White)	3 (1.0)
Native Hawaiian or Other Pacific Islander	0 (0)
Unknown/Decline to state	6 (2.0)
*Training Level*	
Junior residents	171 (57.0)
PGY-1	77 (25.7)
PGY-2	42 (14.0)
PGY-3	52 (17.3)
Research residents	52 (17.3)
Senior residents	75 (25.0)
PGY-4	43 (14.3)
PGY-5	32 (10.7)
Decline to state	2 (0.7)

### Association between resilience/risk factors and flourishing

Descriptive statistics, along with normative data for each measure as available, are reported in Table 2. MHC score was significantly positively correlated with all measured resilience factors and significantly negatively correlated with all measured risk factors (Table 3). In the multivariable model including both resilience and risk factors, we found mindfulness (Std. *β* = 0.03, *p* = 0.0064), personal accomplishment (PA) (Std. *β* = 0.11, *p* < 0.0001), and workplace support (*β* = 0.08, *p* < 0.0001) to be positively and significantly associated with MHC score, with PA having the strongest resilience effect (i.e., highest Std. *β* among resilience factors) on MHC. Depression (Std. *β* = 0.14, *p* < 0.0001) and anxiety (Std. *β* = 0.05, *p* = 0.0004) were negatively and significantly associated with MHC score, with depression having a stronger risk effect (i.e., higher Std. *β*) on MHC than anxiety. Among factors with statistically significant effects, Std. *β* was highest for depression (Std. *β* = 0.14), followed by PA (Std. *β* = 0.11), then workplace support (Std. *β* = 0.08), then anxiety (Std. *β* = 0.05), then mindfulness (Std. *β* = 0.03). Stress was strongly correlated with both depression and anxiety. Therefore, we omitted stress from this model. Comparison of the multivariable model subsample (*n* = 237) and the subsample excluded due to missing data (*n* = 63) did not differ significantly on any of the participant characteristics tested. Consequently, complete case analysis was adequate to produce approximately unbiased estimates.

**Table 2:** Descriptive statistics MHC score and resilience/risk factors, with normative data

Factors	Study Population		Normative Data	
	*N* (%)^a^	Mean (SD)	Mean (SD)	Population (All U.S.)
*Global wellbeing*				
MHC	293 (97.7)	45.89 (13.39)	47.46 (NR)	5689 College students [44]
*Resilience factors*				
CAMS-R	265 (88.3)	27.87 (5.21)	31.51 (5.65)	212 [16, 26]
aMBI-PA	263 (87.7)	13.82 (2.71)	–^b^	
DCSQ-Support	294 (98.0)	19.21 (3.13)	18.34 (2.68)	411 White-collar employees [61]
DCSQ-Control	288 (96.0)	13.73 (2.15)	17.31 (2.84)	411 White-collar employees [61]
*Risk factors*				
PHQ	264 (88.0)	6.65 (4.98)	4.5 (5.5)	704 Parents of school-age children, age 31–40 [75]
aMBI-EE	265 (88.3)	9.95 (4.13)	–^b^	
aMBI-DP	265 (88.3)	6.80 (4.28)	–^b^	
PSS	262 (87.3)	17.61 (6.25)	17.46 (7.31)	433 Population sample, age 25–34 [17]
STAI	263 (87.7)	12.63 (3.66)	10.7 (NR)	503 Adults [55]
DCSQ-Demand	264 (88.0)	15.66 (2.29)	13.27 (2.43)	411 White-collar employees [61]

*MHC* mental health continuum; *CAMS-R* cognitive and affective mindfulness scale-revised; *aMBI-PA* abbreviated Maslach burnout inventory-personal accomplishment; *DCSQ-Support* demand, control, support questionnaire-support; *DCSQ-Control* demand, control, support questionnaire-control; *PHQ* patient health questionnaire (measure of depression); *aMBI-EE* abbreviated Maslach burnout inventory-emotional exhaustion; *aMBI-DP* abbreviated Maslach burnout inventory-depersonalization; *PSS* perceived stress scale; *STAI* state-trait anxiety index; *DCSQ-Demand* demand, control, support questionnaire-demand; *SD* standard deviation; *NR* not reported

^a^Number of respondents of each instrument (% of total 300 respondents)

^b^Normative data not available since instrument was developed and exclusively used for physicians

**Table 3:** Cross-sectional associations between MHC score and resilience/risk factors, unadjusted and multivariable model results

Factors	MHC Score						
	Unadjusted			Adjusted^a^			
	β^c^	SE	*p* value	β^b^	SE	*p* value	Std. β^c^
*Resilience Factors*							
CAMS-R	1.42	0.13	< 0.001	0.32	0.12	0.01	0.03
aMBI-PA	3.07	0.24	< 0.001	1.27	0.23	< 0.001	0.11
DCSQ-support	2.44	0.21	< 0.001	0.91	0.20	< 0.001	0.08
DCSQ-control	2.29	0.35	< 0.001	0.34	0.27	0.20	0.003
*Risk Factors*							
PHQ	− 1.88	0.12	< 0.001	− 0.88	0.14	< 0.001	0.14
aMBI-EE	− 1.67	0.17	< 0.001	− 0.07	0.20	0.74	0.004
aMBI-DP	− 1.08	0.18	< 0.001	0.06	0.16	0.70	0.004
PSS	− 1.48	0.09	< 0.001				
STAI	− 2.28	0.17	< 0.001	− 0.66	0.18	< 0.001	0.05
DCSQ-demand	− 1.42	0.34	< 0.001	0.39	0.24	0.10	0.007

*MHC* mental health continuum; *CAMS-R* cognitive and affective mindfulness scale-revised; *aMBI-PA* abbreviated Maslach burnout inventory-personal accomplishment; *DCSQ-Support* demand, control, support questionnaire-support; *DCSQ-Control* demand, control, support questionnaire-control, *PHQ* patient health questionnaire (measure of depression); *aMBI-EE* abbreviated Maslach burnout inventory-emotional exhaustion; *aMBI-DP* abbreviated Maslach burnout inventory-depersonalization; *PSS* perceived stress scale; *STAI* state-trait anxiety index; *DCSQ-Demand* demand, control, support questionnaire-demand, *SE* standard error

^a^Adjusted for all resilience and risk factors

^b^Linear regression coefficient

^c^Standardized partial omega-square effect size (small 0.01, medium 0.06, large 0.14)

## Discussion

This national cross-sectional study of mixed-PGY trainees at 16 academic General Surgery Residency programs revealed a spectrum of measurable, modifiable individual and workplace factors that appear to impact the prevalence of flourishing (or the magnitude of MHC score) in this population. This suggests potential targets for interventions to enhance flourishing (i.e., global wellbeing or thriving) in surgical trainees. Specifically, our results revealed three key findings: first, that flourishing (a metric with validity evidence as a measure of global wellbeing) is influenced by modifiable individual and workplace factors; second, these modifiable factors have differential impact on MHC score; and third, mindfulness-based cognitive skills training may be the highest yield near-term intervention to improve trainee wellbeing, in spite of its modest relative magnitude impact on flourishing.

Our first finding, that flourishing is influenced by modifiable individual and workplace factors, is evidenced by statistically significant associations with mindfulness, personal accomplishment, depression, anxiety and workplace support. Given the validity evidence for flourishing as a measure global wellbeing in surgical trainees [53], these targetable factors suggest opportunities for intervention. Flourishing, as measured by the MHC, is a widely regarded psychological construct, which represents a conceptualization of wellbeing that encompasses social connectedness (social wellbeing), positive emotions (emotional wellbeing), and positive functioning (psychological wellbeing) [32, 41]. Although prior studies have shown an inverse relationship between flourishing and depressive symptoms [32] and a direct relationship between flourishing and mindfulness [14], flourishing is not simply the absence of mental illness or pathology [43, 47], nor is it the product of a single resource. Rather, flourishing represents the composite of multiple simultaneous individual and environmental factors that comprise global wellbeing (or thriving). As such, we propose flourishing as a meaningful metric for evaluating wellbeing interventions or workplace changes that aim to support the optimal functioning of surgical trainees.

Further, our results reflect two established theories. First, the broaden-and-build theory of positive emotions [28] suggests that flourishers thrive by purposefully cultivating positive emotions (e.g., joy, love, contentment, and interest) through the development and regular use of cognitive habits, such as emotional regulation, objective attention, and meta-cognition (skills specifically trained through mindfulness meditation) [30, 54]. As a consequence, flourishing individuals build more inner and outer resources (such as individual capability and collaborative support [6, 29]) over time [14], thereby cyclically enhancing their confidence, positive outcomes, and the development of collaborative resources. Second, the Job Demand-Resource theory [20] suggests that job strain (which includes burnout) develops in settings where workplace demands outstrip resources [27, 70], and has been shown in other settings to be associated with lower flourishing [11]. Importantly, these two theories are complementary in their insights. Achieving a state of flourishing, though influenced by positive psychology as suggested by the Broaden & Build theory, should not be the sole responsibility of individual physicians or trainees. Similarly, while workplace factors outlined in the Job Demand-Resource theory play an important role, individuals should also be empowered to optimize their own sphere of influence. As such, flourishing represents a theoretically and empirically grounded metric that accounts for the effects of both individual and workplace factors on thriving [24, 38, 51, 62, 83]. This provides us with an objective metric for the critical evaluation of targets and interventions relevant to developing wellbeing programs in surgery. Nuances of these theories have undergone scrutiny in the literature, but in the multiple decades since their introduction [20, 28], the body of empirical evidence supporting the validity and saliency of broaden-and-build and JDR theories have far outweighed scant criticism.

Our second finding, that individual and workplace factors have differential impact on MHC score, is evidenced by the differences in the magnitudes of the effect sizes in our adjusted analysis of influential risk and resilience factors. In terms of individual factors, Maslach Burnout Inventory-Personal Accomplishment (MBI-PA) and Cognitive Affective Mindfulness Scale-Revised (CAMS-R) scores are positively, significantly associated with MHC score, with MBI-PA effect greater than that of CAMS-R. Low PA (one dimension of burnout [58]), has been described as lack of perceived professional efficacy (i.e., feelings of inadequacy and failure) and is thought to be influenced by relationships with superiors and colleagues [58]. This underscores PA being derived from both individuals and the workplace environment and indeed low PA are correlated with attrition from surgical residency [45]. To date, concrete strategies to increase PA for surgical trainees have not been defined thus limiting the feasibility of targeting this potentially high-impact factor in the near term.

The Patient Health Questionnaire (PHQ) and State Trait Anxiety Index (STAI) scores are negatively, significantly associated with MHC score, with PHQ having a higher magnitude effect than STAI, MBI-PA, and CAMS-R. Both depression and anxiety are phenomena that can be addressed through cognitive, behavioral, and/or pharmacologic interventions under the purview of mental health professionals [13, 37]. However, while a majority of residents believe they would benefit from psychiatric care, less than a quarter of those who feel they need treatment actually seek it [1]. Though trainees are at increased risk for developing depression compared to the general population [22], barriers to seeking care include time constraints, prohibitive cost (given low residency pay), and stigma surrounding mental illness in medicine [1, 73]. It is worth noting that efforts to address these barriers may represent one area of opportunity for programs to increase surgical trainee wellbeing, under active exploration at an increasing number of programs.

Mindfulness (as measured by CAMS-R) has a lower magnitude impact on flourishing than PA, PHQ, and STAI, yet evidence exists that surgery residents can be feasibly trained in mindfulness [49, 56] through an available structured curriculum [51] with promising benefits to burnout and physiologic measures of stress. Of note, PA has been shown to increase through mindfulness-based interventions in other settings [18, 39, 57] and mindfulness training has been shown to directly address depression and anxiety [31, 33, 36, 81] in clinical populations. Mindfulness-based interventions are thought to increase one’s ability to evaluate uncomfortable experiences more judiciously, thereby changing one’s relationship to stress and negative thinking by teaching individuals to recognize thoughts, emotions, and sensations [68] with non-reactivity [50]. For example, mindfulness has been shown to help individuals objectively and non-judgmentally identify thoughts, emotions, and sensations underlying the worry patterns that reinforce anxiousness, helping to break the cognitive habits that help perpetuate clinical anxiety [10, 84]. This suggests that cognitive skills-based training focused on interoception (i.e., moment-to-moment situational awareness of thoughts, emotions, and sensations), emotional regulation (i.e., learned nonreactivity in response to these stimuli), and meta-cognition (i.e., utilization of these cognitive skill on demand) may additionally serve as an effective intervention to address stress-based pathologies.

In terms of workplace factors, workplace support was found to be positively, significantly associated with MHC score. The magnitude of effect of workplace support on flourishing exceeded that of mindfulness and anxiety, but was less than PA and depression. Workplace support, as measured by the Demand Support Control Questionnaire (DCSQ), can involve elements, such as workplace atmosphere, employee–employer value congruence (and compatibility), and responsive workplace systems [71]. In an attempt to increase residents’ perceived support, many surgical training programs have begun to implement interventions, such as professional mentorship, social outings, and group counseling sessions [15, 69]. However, to our knowledge, the impact of these specific interventions on objective measures of workplace support or global wellbeing has not been shown, thus limiting our ability to critically evaluate such interventions.

Our third finding, that mindfulness-based cognitive skills training may be the highest yield near-term intervention to improve trainee wellbeing, in spite of its modest relative magnitude impact on flourishing, is supported by the *combination* of the impact and feasibility of this intervention. As seen by our and others’ data, mindfulness training has been linked with increased mindfulness (as seen by increase in CAMS-R), increased PA, reduced anxiety, depression, and burnout [18, 39, 40, 52, 57, 58], all factors associated with enhanced global wellbeing (i.e., ‘flourishing’). Mindfulness-based cognitive training interventions have been feasibly and acceptably delivered to surgical trainees via a codified and time-limited course [48, 49, 56]. Thus, the feasibility and acceptability of mindfulness training, together with its potential to modify many of the factors identified in our work, suggest that mindfulness training may be a high-yield tool for residency programs to improve resident wellbeing in the near term. This finding should be viewed not as a resolution to place the onus of wellbeing on the shoulders of individual trainees, but rather as a short-term strategy to capitalize on existing tools. While other, similarly feasible and potentially even more impactful targets may exist, further investigation is needed to establish evidence-based individual and system-level interventions.

Although potentially promising, our findings should be viewed in the context of several limitations. Our response rate was 34%, which may introduce bias. Thus, national surveys that are linked to mandatory resident evaluations would be the ideal setting to confirm our findings. While we were able to identify important relationships between individual and workplace factors and flourishing, causality cannot be determined from cross-sectional data. Longitudinal evaluation is needed to reinforce our conclusions. Further, our survey did not probe into details of any prior or on-going exposure to mindfulness practices by respondents. As such, we cannot comment on whether mindfulness scores here reflect inherent mindfulness, the product of specific mindfulness training, or both. This warrants further exploration, particularly in light of our third finding. Finally, recognizing that effective interventions in one setting may not be effective in another [34], further qualitative work exploring the effects of local context [8, 49] on implementation strategies is essential and was not covered here.

## Conclusion

Our results suggest that interventions that increase mindfulness, workplace support, and PA, as well as those that decrease depression and anxiety may prove to be particularly beneficial targets to enhance flourishing (i.e., global wellbeing) in surgical trainees. Next steps include performing longitudinal assessment and collecting qualitative data on the effects of culture and context.

## Supplementary Information

Below is the link to the electronic supplementary material.

Supplementary file1 (PDF 75 kb)

Supplementary file2 (DOCX 13 kb)

## Data Availability

The datasets generated and analyzed during the current study are available from the corresponding author on reasonable request.
