# Supplementary material for: Flourishing as a guide to intervention: a national multicenter study of general surgery residents
Source: Global Surg Educ. 2022 Mar 31;1(1):12. doi: 10.1007/s44186-022-00014-3 (PMC8968303; doi:10.1007/s44186-022-00014-3)
Supplement: Supplementary file 1 — Supplementary file1 (PDF 75 kb) [file 44186_2022_14_MOESM1_ESM.pdf]

Appendix 1. Complete survey instrument

DEMOGRAPHICS

Gender **With which gender do you identify?**

Female
Male
Transgender man / Transman
Transgender woman / Transwoman
Genderqueer / Gender nonconforming
Additional identity (please specify)
Decline to state

If you indicated "Another identity" for question above, please specify:

Race/ethnic **With which race/ethnicity do you identify? Select all that apply.**

American Indian or Alaska Native
Asian
Black or African American
Latinx
Native Hawaiian or Other Pacific Islander
White or caucasian
Other (please specify below)
Unknown or decline to answer

If you indicated "Other" for question above, please specify:

Training Le **What is your current level of training?**

PGY-1
PGY-2
PGY-3
PGY-4
PGY-5
Lab/research resident

Mindfulness **Have you received mindfulness-based training through your residency program (e.g., ESRT, CBT or other)?**

No
Yes

Mental Health Continuum (MHC)

During the PAST MONTH how often did you feel…

		Never	Once or twice	About once a week	2 or 3 times a week	Almost every day	Every day
MHC 1	Happy:						
MHC 2	Interested in life:						
MHC 3	Satisfied with life:						
MHC 4	That you had something important to contribute to society:						
MHC 5	That you belonged to a community (like a social group, school, neighborhood):						
MHC 6	That our society is a good place, or is becoming a better place, for all people:						
MHC 7	That people are basically good:						
MHC 8	That the way our society works made sense to you:						
MHC 9	That you liked most parts of your personality:						
MHC 10	Good at managing the responsibilities of your daily life:						
MHC 11	That you had warm and trusting relationships with others:						
MHC 12	That you had experiences that challenged you to grow and become a better person:						
MHC 13	Confident to express your own ideas and opinions:						
MHC 14	That your life has a sense of direction or meaning to it:						

Demand Control Support Questionnaire (DCSQ)

For each item, please respond with the answer choice that best describes you:

		Strongly disagree	Disagree	Agree	Strongly Agree
DCSQ 1	There is a quiet and/or pleasant atmosphere at my place of work				
DCSQ 2	There is good collegiality at work				
DCSQ 3	My colleagues support me				
DCSQ 4	People at work understand that I may have a bad day				
DCSQ 5	I get along well with my superiors				
DCSQ 6	I get along well with my colleagues				
DCSQ 7	Does your job require you to work very fast?				
DCSQ 8	Does your job require you to work very hard?				
DCSQ 9	Does your job require too great a work effort?				
DCSQ 10	Do you have sufficient time for all your work tasks?				
DCSQ 11	Do conflicting demands often occur in your work?				
DCSQ 12	Do you have the opportunity to learn new things in your work?				
DCSQ 13	Does your job require creativity?				
DCSQ 14	Does your job require doing the same tasks over and over again?				
DCSQ 15	Do you have the possibility to decide for yourself how to carry out your work?				
DCSQ 16	Do you have the possibility to decide for yourself what should be done in your work?				

Cognitive and Affective Mindfulness Scale-Revised (CAMS-R)

For each item, please respond with the answer choice that best describes you:

		Not at all	Sometimes	Often	Almost always
CAMSR 1	It is easy for me to concentrate on what I am doing:				
CAMSR 2	I can tolerate emotional pain:				
CAMSR 3	I can accept things I cannot change:				
CAMSR 4	I can usually describe how I feel at the moment in considerable detail:				
CAMSR 5	I am easily distracted:				
CAMSR 6	It's easy for me to keep track of my thoughts and feelings:				
CAMSR 7	I try to notice my thoughts without judging them:				
CAMSR 8	I am able to accept my thoughts and feelings:				
CAMSR 9	I am able to focus on the present moment:				
CAMSR 10	I am able to pay close attention to one thing for a long period of time:				

Abbreviated Maslach Burnout Inventory (aMBI)

For each item, please state how often you feel this way about the work you do:

		Never	A few times a year or less	Once a month or less	A few times a month	Once a week	A few times a week	Every day
MBI 1	I deal very effectively with the problems of my patients							
MBI 2	I feel I treat some patients as if they were impersonal objects							
MBI 3	I feel emotionally drained from my work							
MBI 4	I feel fatigued when I get up in the morning and have to face another day on the job							
MBI 5	I have become more callous toward people since I took this job							
MBI 6	I feel I'm positively influencing other people's lives through my work							
MBI 7	Working with people all day is really a strain for me							
MBI 8	I don't really care what happens to some patients							
MBI 9	I feel exhilarated after working closely with my patients							

Patient Health Questionnaire (PHQ)

Over the last 2 WEEKS, how often have you been bothered by any of the following problems?

		Not at all	Several days	More than half the days	Nearly every day
PHQ 1	Little interest or pleasure in doing things?				
PHQ 2	Feeling down, depressed or hopeless?				
PHQ 3	Trouble falling or staying asleep, or sleeping too much				
PHQ 4	Feeling tired or having little energy				
PHQ 5	Poor appetite or overeating				
PHQ 6	Feeling bad about yourself - or that you are a failure or have let yourself or your family down				
PHQ 7	Trouble concentrating on things, such as reading the newspaper or watching television				
PHQ 8	Moving or speaking so slowly that other people could have noticed. Or the opposite - being so fidgety or restless that you have been moving around a lot more than usual.				

Percieved Stress Scale (PSS)

In the LAST MONTH, how often have you…

		Never	Almost never	Sometimes	Fairly often	Very often
PSS 1	...how often have you been upset because of something that happened unexpectedly?					
PSS 2	...how often have you felt that you were unable to control the important things in you life?					
PSS 3	...how often have you felt nervous and "stressed"?					
PSS 4	...how often have you felt confident about your ability to handle your personal problems?					
PSS 5	...how often have you felt that things were going your way?					
PSS 6	...how often have you found that you could not cope with all the things you had to do?					
PSS 7	...how often have you been able to control irritations in your life?					
PSS 8	...how often have you felt that you were on top of things?					
PSS 9	...how often have you been angered because of things that were outside of your control?					
PSS 10	...how often have you felt difficulties were piling up so high that you could not overcome them?					

Stait-Trait Anxiety Inventory (STAI)

Please respond to the following statements for how you feel NOW:

		Not at all	Somewhat	Moderately so	Very much so
STAI 1	...I feel calm				
STAI 2	...I feel tense				
STAI 3	...I feel upset				
STAI 4	...I feel relaxed				
STAI 5	...I feel content				
STAI 6	...I feel worried				
