# Supplementary material for: Flourishing as a guide to intervention: a national multicenter study of general surgery residents
Source: Global Surg Educ. 2022 Mar 31;1(1):12. doi: 10.1007/s44186-022-00014-3 (PMC8968303; doi:10.1007/s44186-022-00014-3)
Supplement: Supplementary file 2 — Supplementary file2 (DOCX 13 kb) [file 44186_2022_14_MOESM2_ESM.docx]

**Appendix 2**. Mental Health Continuum questions

| **MHC Item**  *During the past month how often did you feel...* | Domain |
| --- | --- |
|  |  |
| **MHC 1**  ...Happy | Emotional Wellbeing |
| **MHC 2**  ...Interested in life | Emotional Wellbeing |
| **MHC 3**  ...Satisfied with life | Emotional Wellbeing |
| **MHC 4**  ...That you had something important to contribute to society | Social Wellbeing |
| **MHC 5**  ...That you belonged to a community (like a social group, school, neighborhood) | Social Wellbeing |
| **MHC 6**  ...That our society is a good place, or is becoming a better place, for all people | Social Wellbeing |
| **MHC 7**  ...That people are basically good | Social Wellbeing |
| **MHC 8**  ...That the way our society works made sense to you | Social Wellbeing |
| **MHC 9**  ...That you liked most parts of your personality | Psychological Wellbeing |
| **MHC 10**  ...Good at managing the responsibilities of your daily life | Psychological Wellbeing |
| **MHC 11**  ...That you had warm and trusting relationships with others | Psychological Wellbeing |
| **MHC 12**  ...That you had experiences that challenged you to grow and become a better person | Psychological Wellbeing |
| **MHC 13**  ...Confident to express your own ideas and opinions | Psychological Wellbeing |
| **MHC 14**  ...That your life has a sense of direction or meaning to it | Psychological Wellbeing |
| MHC = Mental Health Continuum | |
